# “Ground-Truthing” Efficacy of Biological Control for Aflatoxin Mitigation in Farmers’ Fields in Nigeria: From Field Trials to Commercial Usage, a 10-Year Study

**DOI:** 10.3389/fmicb.2019.02528

**Published:** 2019-11-08

**Authors:** Ranajit Bandyopadhyay, Joseph Atehnkeng, Alejandro Ortega-Beltran, Adebowale Akande, Titilayo D. O. Falade, Peter J. Cotty

**Affiliations:** ^1^International Institute of Tropical Agriculture, Ibadan, Nigeria; ^2^International Institute of Tropical Agriculture, Abuja, Nigeria; ^3^Agricultural Research Service, United States Department of Agriculture, Tucson, AZ, United States

**Keywords:** aflatoxin, biocontrol, efficacy trials, long-term efficacy, maize, groundnut, safe food

## Abstract

In sub-Saharan Africa (SSA), diverse fungi belonging to *Aspergillus* section *Flavi* frequently contaminate staple crops with aflatoxins. Aflatoxins negatively impact health, income, trade, food security, and development sectors. *Aspergillus flavus* is the most common causal agent of contamination. However, certain *A. flavus* genotypes do not produce aflatoxins (i.e., are atoxigenic). An aflatoxin biocontrol technology employing atoxigenic genotypes to limit crop contamination was developed in the United States. The technology was adapted and improved for use in maize and groundnut in SSA under the trademark Aflasafe. Nigeria was the first African nation for which an aflatoxin biocontrol product was developed. The current study includes tests to assess biocontrol performance across Nigeria over the past decade. The presented data on efficacy spans years in which a relatively small number of maize and groundnut fields (8–51 per year) were treated through use on circa 36,000 ha in commercially-produced maize in 2018. During the testing phase (2009–2012), fields treated during one year were not treated in the other years while during commercial usage (2013–2019), many fields were treated in multiple years. This is the first report of a large-scale, long-term efficacy study of any biocontrol product developed to date for a field crop. Most (>95%) of 213,406 tons of maize grains harvested from treated fields contained <20 ppb total aflatoxins, and a significant proportion (>90%) contained <4 ppb total aflatoxins. Grains from treated plots had preponderantly >80% less aflatoxin content than untreated crops. The frequency of the biocontrol active ingredient atoxigenic genotypes in grains from treated fields was significantly higher than in grains from control fields. A higher proportion of grains from treated fields met various aflatoxin standards compared to grains from untreated fields. Results indicate that efficacy of the biocontrol product in limiting aflatoxin contamination is stable regardless of environment and cropping system. In summary, the biocontrol technology allows farmers across Nigeria to produce safer crops for consumption and increases potential for access to premium markets that require aflatoxin-compliant crops.

## Introduction

Throughout sub-Saharan Africa (SSA), certain *Aspergillus* species frequently contaminate with aflatoxins several staple crops, including maize and groundnut (Shephard, 2008; Udomkun et al., 2017). In SSA, human and animal aflatoxin exposure is high (JECFA, 2018; Sirma et al., 2018; Blankson et al., 2019). Consumption of highly contaminated food can result in acute health effects such as liver diseases and death (Gieseker, 2004; Probst et al., 2010; Kamala et al., 2018). Chronic, sub-lethal exposure may cause child stunting, immunosuppression, impaired food conversion, and cancer (Coursaget et al., 1993; Gong et al., 2008; JECFA, 2018; Leroy et al., 2018; Voth-Gaeddert et al., 2018). Trade sectors also become affected. High aflatoxin content restricts farmers’ access to local and international premium markets. This results in reduced income for farmers but also aggregators, processors, and exporters (Williams, 2008; Wu, 2015). Because of the challenges posed by aflatoxins, substantial efforts have been made to both understand the contamination process and design management programs to reduce food safety risks (James et al., 2007; Hell et al., 2008; Bandyopadhyay et al., 2016; Seetha et al., 2017).

Aflatoxin-producing *Aspergillus* species of agricultural importance belong to section *Flavi* (Frisvad et al., 2019). Some species produce only B aflatoxins while others produce both B and G aflatoxins. There are four major aflatoxins (B_1_, B_2_, G_1_, G_2_) with aflatoxin B_1_ both the most toxic and prevalent and classified as a Group 1 carcinogen by the International Agency for Research on Cancer (IARC, 2002; JECFA, 2018). The major causal agent of contamination is *A. flavus*, which produces only B aflatoxins (Amaike and Keller, 2011). *A. flavus* is composed of the L and S morphotypes, which differ in morphological, physiological, and genetic criteria (Cotty, 1989). Each morphotype can be further subdivided in numerous vegetative compatibility groups (VCGs) (Bayman and Cotty, 1991; Ortega-Beltran and Cotty, 2018). Genetic variation within members of a VCG is low because they descend from a single clonal lineage but members of different VCGs vary in several characters (Leslie, 1993; Grubisha and Cotty, 2010, 2015). Most L morphotype fungi produce aflatoxins (at varying levels) while others produce no aflatoxins (i.e., are atoxigenic) due to lesions in the aflatoxin biosynthesis gene cluster (Adhikari et al., 2016). Furthermore, there are L morphotype VCGs composed exclusively of atoxigenic members (Mehl et al., 2012; Grubisha and Cotty, 2015; Atehnkeng et al., 2016).

Species other than *A. flavus* may be important etiologic agents of aflatoxin contamination. In West Africa, fungi resembling the *A. flavus* S morphotype but that produce both B and G aflatoxins are associated with several crops, including maize and groundnut (Cotty and Cardwell, 1999; Atehnkeng et al., 2008a; Donner et al., 2009; Diedhiou et al., 2011; Agbetiameh et al., 2018; Ezekiel et al., 2019). These fungi were previously known as unnamed taxon S_BG_. Fungi in this group may be any of recently described species that include *A. aflatoxiformans*, *A. austwickii*, *A. cerealis*, *A. minisclerotigenes*, and unnamed taxa (Probst et al., 2014; Frisvad et al., 2019; Singh and Cotty, 2019). As in a previous study (Ezekiel et al., 2019), in the current study we use the term S_BG_ strains for all fungi with S morphotype producing both B and G aflatoxins. Even though S_BG_ strains are found at low levels in some years/crops, the extremely high aflatoxin-producing potential of the group warrants special consideration as a major contributor to aflatoxin contamination events in West Africa.

Aflatoxin management strategies have been sought for more than 40 years. Most pre- and post-harvest strategies may reduce incidences and severities of aflatoxin contamination (Bruns, 2003; Cotty et al., 2008; Hell et al., 2008; Waliyar et al., 2015; Seetha et al., 2017; Mahuku et al., 2019). However, the use of a single management strategy in isolation may not prevent initiation of aflatoxin contamination and be insufficient to reduce aflatoxin contamination to acceptable levels [i.e., at least below 20 parts per billion (ppb)] (Bandyopadhyay et al., 2016). Therefore, aflatoxin management strategies must address the contamination process throughout crop production and until crops are consumed using holistic interventions (Ayalew et al., 2017; Logrieco et al., 2018).

The most promising strategy to control aflatoxins is the use of atoxigenic *A. flavus* strains to competitively displace aflatoxin producers (Amaike and Keller, 2011). This strategy favors the prevalence of atoxigenic strains in the treated fields and throughout the environment. When less aflatoxin producers are associated with a crop, less aflatoxins accumulate (Mehl et al., 2012). This aflatoxin management strategy protects crops from the field, throughout storage, and until consumption. The US Department of Agriculture – Agricultural Research Service (USDA-ARS) developed the first atoxigenic biocontrol product, *Aspergillus flavus* AF36, and initially registered it with the US Environmental Protection Agency (USEPA) for use in cotton (USEPA, 2003, 2004). Together with Afla-Guard^®^, a second atoxigenic biocontrol product, the biocontrol technology has been used commercially for >15 years in the US in several crops (Cotty et al., 2007; Dorner, 2009; Doster et al., 2014; Ortega-Beltran and Bandyopadhyay, 2019).

In 2003, the International Institute of Tropical Agriculture (IITA) started a collaboration with USDA-ARS to adapt and improve the biocontrol technology for use in Nigeria and subsequently in other SSA nations (Bandyopadhyay et al., 2016). Initial efforts in Nigeria included examining fungal communities associated with maize and field soils and assessing aflatoxin-producing potentials of the fungi (Atehnkeng et al., 2008a; Donner et al., 2009). Distribution and frequencies of atoxigenic genotypes across target environments in Nigeria were used as criteria to select those atoxigenic African *Aspergillus flavus* VCGs (AAVs) with superiority in adaptation, fitness, and competitiveness (Atehnkeng et al., 2016). In parallel, studies to determine genetic lesions causing atoxigenicity in atoxigenic AAVs were conducted (Donner et al., 2010). Moreover, representative isolates of selected atoxigenic AAVs were evaluated under laboratory conditions (Atehnkeng et al., 2008b), and in farmers’ fields across Nigeria to further select competitive and widely adapted atoxigenic AAVs (Atehnkeng et al., 2014). Four superior isolates, each representing a unique AAV composed only of atoxigenic members, are the active ingredient fungi of the biocontrol product Aflasafe^®^ (hitherto called ‘biocontrol product’). In 2014, Nigeria’s National Agency for Food and Drug Administration and Control (NAFDAC) approved the full registration of the biocontrol product for unrestricted use in both maize and groundnut across Nigeria (Bandyopadhyay et al., 2016).

Several technologies aiming to improve crop productivity and/or quality—including technologies to reduce crop aflatoxin content—are often evaluated in experimental stations, or on farmer fields managed by researchers, and typically in a few fields (Alaniz Zanon et al., 2013; Weaver et al., 2015; Accinelli et al., 2018; Molo et al., 2019; Weaver and Abbas, 2019). Evaluating technologies under farmer field conditions, in multiple fields of multiple agro-ecological zones (AEZ), and in multiple years, under real-life situations that farmers face can aid in drawing unbiased conclusions about a technology’s efficacy (Ortega-Beltran and Bandyopadhyay, 2019). Multi-year, multi-location evaluations are needed to determine if a technology is valuable to farmers. Indeed, this important biocontrol technology has been often questioned and one of the main concerns is that multi-year, multi-area studies are needed to clarify whether aflatoxin management using atoxigenic fungi is a valuable tool, particularly in African contexts (Ehrlich et al., 2015; Njoroge, 2018; Stepman, 2018; Pitt, 2019; Kagot et al., 2019).

The current study reports the longest-term efficacy study of any aflatoxin biocontrol product developed to date. We present results of biocontrol product efficacy across Nigeria at first in (i) field trials in maize and groundnut farmers’ fields (2009–2012), and then in (ii) commercially-produced maize (2013–2018). Post-harvest benefits of using pre-harvest biocontrol are discussed as well. The results obtained during these 10 years demonstrate that biocontrol provides stable aflatoxin reductions and is a valuable aflatoxin mitigation tool used by maize and groundnut farmers across Nigeria regardless of their farming practices, crop varieties, or environmental challenges. Biocontrol allows farmers in Nigeria to produce maize and groundnut with aflatoxin concentrations safe for consumption and trade.

## Materials and Methods

### The Active Ingredient Fungi of the Biocontrol Product

The biocontrol product contains four atoxigenic *A. flavus* L morphotype isolates (Ka16127, La3304, La3279, and Og0222) that belong to atoxigenic AAVs native and relatively common in the major maize and groundnut producing areas of Nigeria (Atehnkeng et al., 2014, 2016). Ka16127 was recovered from maize produced in Saminaka, Kaduna state; La3304 and La3279 were recovered from maize cropped in Lafia, Nasarawa state; Og0222 was recovered from maize cropped in Ogbomosho, Oyo state (Atehnkeng et al., 2016). The four strains are maintained in the fungal collection of both IITA and USDA-ARS as sporulating cultures in water vials and silica grain vials for short- and long-term storage, respectively.

### The Treated Fields

The biocontrol product was applied in maize and groundnut farmer field trials (2009–2012) and in commercial maize (maize produced for commercial markets) fields (2013–2018) (Table 1). All fields belonged to smallholder farmers. Cropping systems, climatic conditions, maize and groundnut cultivars used by farmers, disease and pest pressure, among other factors were variable across the different areas of Nigeria where the biocontrol product was used. Results from field efficacy trials conducted in 2009 and 2010 were used to prepare a dossier for registration of the biocontrol product with NAFDAC (Bandyopadhyay et al., 2016). Trials conducted in 2011 and 2012 were used to demonstrate to regulators efficacy of the product under additional conditions and to create market-linkages with poultry and food industries seeking aflatoxin-compliant crops. Commercial maize fields treated with the biocontrol product during 2013–2018 were part of the AgResults Nigeria Aflasafe^TM^ Challenge Project, hitherto called as the ‘AgResults Project’ (Bandyopadhyay et al., 2016; AgResults, 2019; Schreurs et al., 2019).

**TABLE 1:** Number of maize and groundnut samples from fields treated with an aflatoxin biocontrol product and accompanying untreated fields (field efficacy trials, 2009–2012) and samples taken from commercially treated and control maize (2013–2018).

**Year**	**Crop**	**Purpose**	**Number of samples^z^**	
			
			**Biocontrol-treated**	**Control**
2009	Maize	Farmer field efficacy trials	51	51
	Groundnut	Farmer field efficacy trials	8	8
2010	Maize	Farmer field efficacy trials	14	14
	Groundnut	Farmer field efficacy trials	16	16
2011	Maize	Development of market linkages	199	199
	Groundnut	Development of market linkages	82	82
2012	Maize	Development of market linkages	38	38
2013	Maize	Commercial use by farmers	660	0
2014	Maize	Commercial use by farmers	213	99
2015	Maize	Commercial use by farmers	292	109
2016	Maize	Commercial use by farmers	1,314	0
2017	Maize	Commercial use by farmers	2,451	257
2018	Maize	Commercial use by farmers	2,751	240

*^*z*^During 2009–2013, grain samples were collected from individual farmer’s field. During 2014–2018, field officers of the AgResults Project collected a 5-kg sample from each 30-ton grain lots aggregated from multiple farmers by commercial enterprises. For example, in 2014, 213 maize samples represent a total of 6,390 tons of biocontrol-treated maize.*

### Biocontrol Product Manufacturing

For the field trials (2009–2012), the biocontrol product was produced using a laboratory-scale method described earlier (Cotty et al., 2007; Atehnkeng et al., 2014). Briefly, spore suspensions of each of the four atoxigenic isolates were harvested from 5-day-old cultures on 5-2 agar [5% V8 juice (Campbell Soup Company, Camden, NJ, United States), 2% Bacto-agar (Difco Laboratories Inc., Detroit, MI, United States), pH 5.2 (Cotty, 1994)] using 0.1% TWEEN^®^ 80. Suspensions were adjusted to a concentration of 10^6^ spores ml^–1^ using a hemocytometer. White sorghum grains were soaked in water for 2 h, drained, and autoclaved for 45 min in polyethylene bags (45 cm × 20 cm). Batches of 1-kg of grain were seeded independently with 100 ml of spore suspensions of each atoxigenic active ingredient isolate, mixed to spread the suspension uniformly, and incubated for 18 h at 31°C. Then, grains were dried at 55°C for 4 days to stop fungal growth. Several batches were prepared for each active ingredient isolate. The final product was formulated by combining batches of 2.5 kg of each isolate into a polyethylene bag and mixed by hand shaking. Product in the polyethylene bags were placed within 10-kg-capacity plastic containers, sealed, and stored at room temperature until use.

The biocontrol product applied in commercial maize fields (2013–2018) was produced in the Aflasafe Manufacturing Plant in IITA-Ibadan (Bandyopadhyay et al., 2016). Briefly, spores of the four atoxigenic isolates were obtained as mentioned above for field trials, and each isolate was multiplied separately in glass bottles containing sterilized sorghum grains that were pre-conditioned before in sterile 1-l plastic bottles. The pre-conditioning process raised the moisture content of sorghum grain to 30% by adding sterile distilled water to the bottles, which were subsequently rolled for 4 h on a 240 Vac Benchtop Roller (Wheaton, Millville, NJ, United States). Pre-conditioned grain (30 g) were added to 250-ml glass bottles along with two Teflon balls (1/2′′ dia) and autoclaved (20 min, 121°C). Each cooled bottle containing sorghum was independently inoculated with 4 ml of spore suspension of each atoxigenic isolate. After incubation (7 days, 31°C), 125 ml sterile 0.1% TWEEN^®^ 80 was added to each bottle to harvest spores. Bottles were placed on a Roto-Shake Genie reciprocal shaker (Scientific Industries, Bohemia, NY, United States) at 200 rpm for 20 min. The Teflon balls helped to dislodge spores from sorghum grains. For each atoxigenic isolate, a suspension was adjusted to 4 × 10^7^ spores ml^–1^ using an Orbeco-Helling digital direct reading turbidimeter (Orbeco Analytical Systems Inc., Farmingdale, NY, United States) and a nephelometric turbidity unit (NTU) vs. CFU (colony-forming units) standard curve (*y* = 49,937*x*; *x* = NTU; *y* = spores ml^–1^). To prepare 100 kg of the product, a spore suspension (1 l, 4 × 10^7^ spores ml^–1^) of the constituent atoxigenic isolates were individually combined with 150 ml of a polymer (Sentry^TM^, Precision Laboratories, Waukegan, IL, United States) and 200 ml of a blue non-toxic dye (Prism^TM^, Milliken and Company, Spartanburg, SC, United States), and coated on roasted, sterile sorghum grain with a seed treater (Bandyopadhyay et al., 2016).

### Quality of the Biocontrol Product

Samples from biocontrol product batches produced either in the laboratory or the manufacturing plant were examined. For each sample, 100 sorghum grains were plated onto two plates each of 5-2 agar, Nutrient Agar (Lab M, United Kingdom; 28 g l^–1^, 20 g l^–1^glucose), and Violet Red Bile Agar (VRBA; Difco Laboratories, 41.5 g l^–1^, pH 7.4). After incubation (7 days, 31°C) plates were examined to count numbers of grains colonized by *A. flavus* and to detect presence or absence of any other microorganism, including fecal coliforms on VRBA. Spore yield was evaluated by placing 24 grains from each batch in individual wells of 24-well cell culture plates, in triplicate, and incubated as above. After incubation, three replicates of two randomly selected grains were rinsed three times with 10 ml 100% ethanol. The resulting wash from each replicate was mixed with 10 ml distilled water and transferred into a turbidimeter vial. Spore yield was quantified by turbidity as above.

Frequencies of the atoxigenic AAVs to which the four active ingredient isolates belong to were determined in all biocontrol product batches. Microbial isolations were done following protocols previously described (Atehnkeng et al., 2008a; Agbetiameh et al., 2018). *Aspergillus* isolates were characterized and saved as previously described (Agbetiameh et al., 2018; Ezekiel et al., 2019). Mutants of *A. flavus* isolates (*nit*) were generated and saved as previously described (Atehnkeng et al., 2014, 2016). Assignment of isolates to one of the four atoxigenic AAV ingredients of the biocontrol product was conducted by performing vegetative compatibility analyses (VCA). In VCA, fungal suspensions of each atoxigenic AAV tester pair and a *nit* mutant being evaluated were individually seeded into wells, spaced by 1 cm in a triangular pattern, in starch media (36 g l^–1^ dextrose, 20 g l^–1^ soluble starch, 3 g l^–1^ NaNO_3_, 20 g l^–1^ Bacto-agar, pH 6.0) (Cotty and Taylor, 2003). Complementary *nit* mutants produce regions of prototrophy indicating restoration of a functional nitrate reductase enzyme (Leslie, 1993). Complementation occurs only between *nit* mutants isogenic at loci governing vegetative incompatibility. *Nit* mutants complementing a tester pair were assigned to the atoxigenic AAV defined by that tester pair.

### Farmers’ Fields

#### Efficacy Trials

**FIGURE 1:**
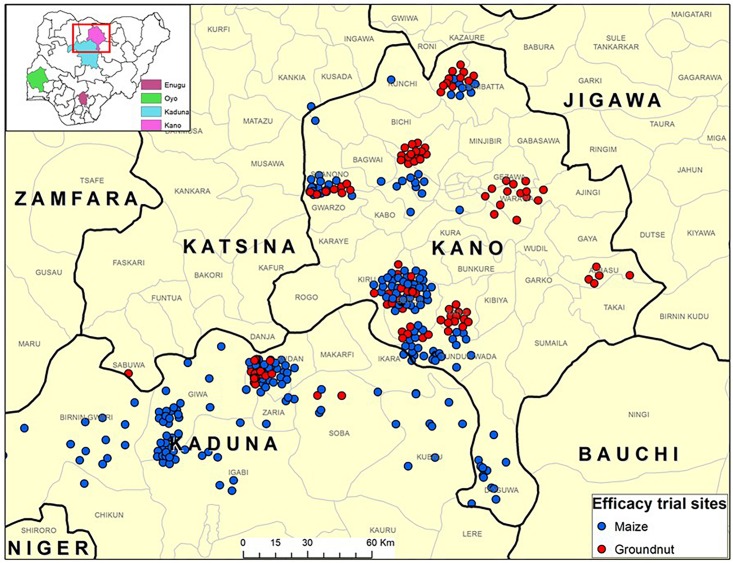
Map of various states of Nigeria (inset) where efficacy trials of an atoxigenic biocontrol product were conducted by maize and groundnut farmers for aflatoxin mitigation during 2009 to 2012. Majority of the trials were conducted in Kano and Kaduna states while a few were conducted in Enugu and Oyo states (inset).

During 2009–2012, efficacy of the biocontrol product was tested by smallholder farmers who voluntarily consented to participate in the experiments in Enugu North, Isi-Uzo, Oji River, and Uzo-Uwani (Enugu state); Birnin-Gwari, Lere, Maigana, Giwa, Soba, and Ikara (Kaduna state); Gwarzo, Tudun Wada, Tsanyanwa, Danbata, Rinin Gado, Rano, Dawakin, Tofa, Albasu, Doguwa, and Warawa (Kano state); and Ogbomosho (Oyo state) (Figure 1). All participating farmers along with agricultural extension agents and trainers were made aware of aflatoxins and their impact (e.g., what aflatoxins are, occurrence in crops, impact on health and trade) and trained on use of biocontrol and other management practices. The on-farm efficacy of the biocontrol product was evaluated using paired plot experimental design (Supplementary Table 1). For each treated maize and groundnut field, there was an accompanying untreated control field of the same crop separated by at least 500 m to avoid interference from movement of biocontrol isolates from treated to control fields (Bock et al., 2004). To avoid carryover influence of inoculum from 1 year to the next, no treated field used in any year was used in subsequent years. Willingness of farmers to participate in the efficacy trials and the crops grown by them also determined the site of the treated and untreated fields. The size of fields ranged from 0.25 to 5.0 ha. Due to interplot interference from movement of inoculum from treated area to control area in small plot settings (Weaver and Abbas, 2019), it was not possible to replicate treatments several times within the small-sized farmer’s fields. Instead, an entire farmer’s field was considered as an experimental unit (replicate) and was either treated or untreated. The number of replicates varied from year to year and are given in Table 1. Farmers grew their crops following their own agronomic practices. In general, farmers’ fields were weeded, earthed-up (i.e., piling up soil around the base of the plants), and top-dressed with urea prior to application of the biocontrol product. Within 15 days post biocontrol product application, no other agronomic intervention was made to avoid burying the product. All fields were rain-fed. The number of treated and control fields for each year is given in Table 1. The biocontrol product was uniformly tossed across the field by hand, at a rate of 10 kg ha^–1^. Prior to application, areas of fields were measured using Garmin eTrex GPS units (Garmin Ltd., Olathe, KS, United States) to determine the quantity of product required to treat individual fields. Extension agents, trainers, and/or IITA staff supervised biocontrol product application.

#### Commercial Use

**FIGURE 2:**
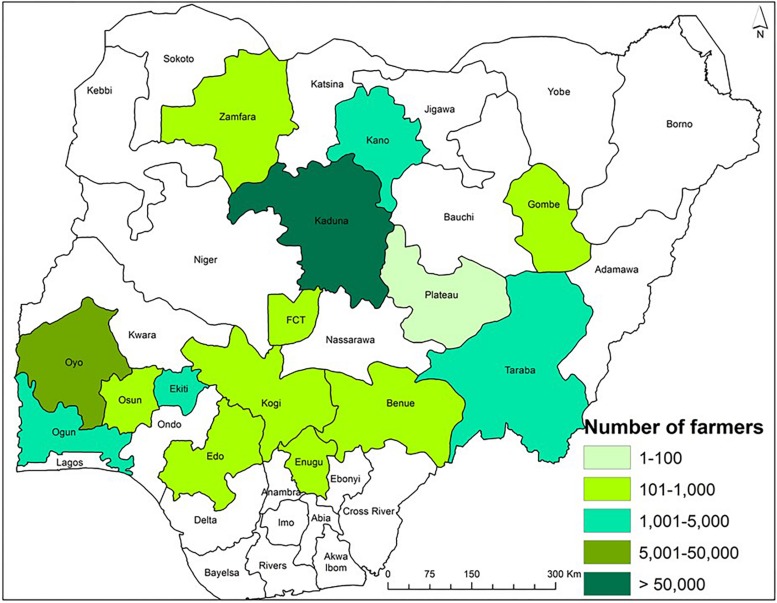
Map of Nigeria showing the number of farmers that participated in the AgResults Project and applied an atoxigenic biocontrol product in maize in various states during 2013 to 2018.

**FIGURE 3:**
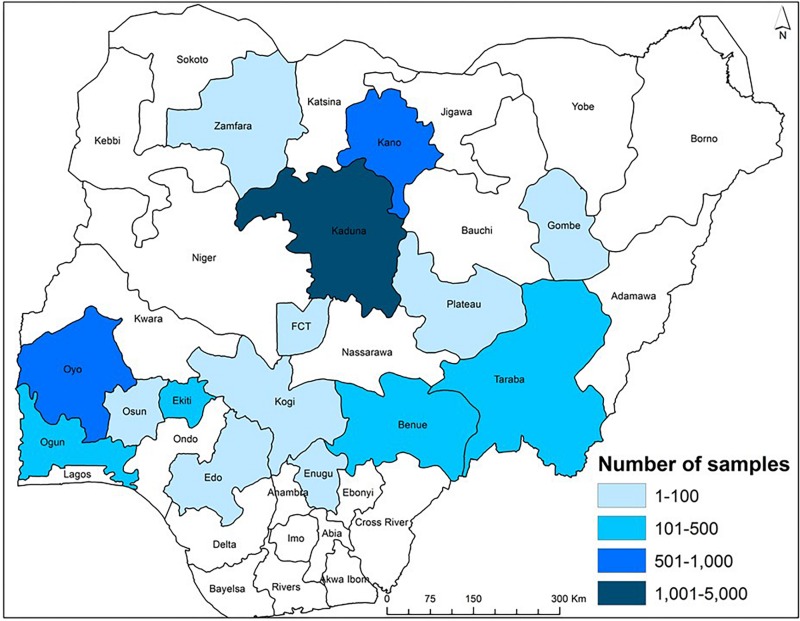
Map of Nigeria showing the range in the number of maize samples collected from aggregation points in various states during 2013 to 2018. Each sample represents approximately a 30-ton grain lot aggregated from farmers who used an atoxigenic biocontrol product on a commercial basis.

During 2013–2018, biocontrol was used only in commercial maize fields by farmers and implementers of the AgResults Project (Bandyopadhyay et al., 2016; Schreurs et al., 2019). Briefly, implementers were private (mostly) or public sector enterprises that worked with groups of smallholder farmers and enabled them to produce biocontrol-treated maize. Implementers purchased and distributed the biocontrol product to farmers and had mutually agreed cost-recovery and profit-sharing arrangements. Implementers were selected by the AgResults Project based on several criteria: (i) ability to organize and coordinate smallholder farmers throughout the cropping season, (ii) ability to provide extension services and access to farm inputs, (iii) possess downstream market linkages to efficiently aggregate and sell quality crops at a premium, and (iv) committed to maximizing transparency, disclosing records, and document-sharing of premium payments or other benefits to their participating farmers, among others. Implementers were trained on improved maize production practices and both pre- and post-harvest aflatoxin mitigation strategies, including use of biocontrol. Once trained, the implementers passed the acquired knowledge to their farmers. During the project, in any given year, some implementers worked with less than 100 farmers while others worked with over 9,000 farmers (Figure 2). The biocontrol product was applied in thousands of fields in the states of Benue, Edo, Ekiti, Enugu, Gombe, Kaduna, Kano, Kogi, Ogun, Osun, Oyo, Plateau, Taraba, Zamfara, and the Federal Capital Territory (FCT) (Figure 3). It is likely that several farmers applied the product in the same field for more than 1 year leading to an unknown extent of carryover effect of biocontrol inoculum from 1 year to the next. As much as possible, samples from non-treated maize were collected from fields about 500 m away from those of participating farmers to enable comparison of paired treated and non-treated samples. In contrast to the 2009–2012 period, the number of control samples was lower than the number of treated samples in 2014, 2015, 2017, and 2018 (Table 1). Control samples were not collected in 2013 and 2016.

### Sampling and Sample Treatment

In both 2009 and 2010, soils of all treated and control fields were collected prior to biocontrol application. Soil from the top 2-cm layer were collected by sub-sampling (∼50 times) from three random locations within each field (Cotty, 1997). Composite samples were placed in cotton bags inside sealed plastic bags and transported to IITA-Ibadan Pathology laboratory. Samples were dried at 48°C for 48 h. Following elimination of soil clods in sterile mortars, samples were manually homogenized within the bags, and then stored at 4°C until subjected to further analyses.

During 2009–2012, 25 maize ears were randomly harvested by hand from treated and control fields. The ears were placed in paper bags and transported to the laboratory. Aflatoxins were quantified on two sets of grain: (i) grain immediately after harvest (15 ears) and (ii) grain subjected to simulated poor storage where 10 ears with husks were wetted for 4 h and allowed to dry slowly over a 10-day period, followed by drying at 48°C for 48 h. Ears from each set were husked and shelled for aflatoxin analysis (Atehnkeng et al., 2014). Sub-samples (500 g) were ground using a blender (Waring Commercial, Springfield, MO, United States) for 1 min in a 250 ml stainless steel blending jar (MC-2), which was decontaminated between samples with 80% ethanol to avoid cross-contamination. Blended and unblended grain samples were kept at 4°C until analyzed. During 2009–2011, groundnut was randomly harvested by hand from treated and control fields. Five kg of unshelled groundnut were placed in paper bags and transported to the laboratory. Aflatoxins were quantified on two sets of grains: (i) grain immediately after harvest (4 kg, 2009–2011) and (ii) grain (1 kg, 2010–2011) subjected to the simulated poor storage conditions, as described for maize. Pods from each set were manually shelled inside a biological safety cabinet and dried as above. Milling and storage of samples was done as above.

For maize from commercial fields (2013–2018), farmers harvested the ears and the implementers aggregated the maize in their stores. Some implementers assisted farmers with threshing, bagging, labeling, and transporting the maize to aggregation stores. At the aggregation point, maize bags (containing around 100 kg maize) were tagged with farmers’ details and arranged according to the quantity supplied by each farmer. Since it was not possible to quantify aflatoxin concentration in individual fields of the >90,000 farmers, grain samples for each 30 tons of treated maize were examined, except for 2013 when samples were collected from individual farmers’ fields. Field officers of the AgResults Project collected a 5-kg sample for each 30 tons of aggregated maize by randomly sampling 50 g of maize from each of 100 bags. The samples were transported to IITA-Ibadan, where they were homogenized, milled, and stored as above.

### Densities of Aflatoxin-Producing Fungi in Soil Before Biocontrol Product Application and Grain at Harvest

These analyses were conducted for 2009 and 2010 biocontrol field efficacy trials. *Aspergillus* section Flavi fungi were obtained from soil and grain by dilution plate technique on Modified Rose Bengal Agar (MRBA) (Cotty, 1994). Briefly, 1 g sample was suspended in 10 ml sterile distilled water contained in 40 ml glass vials and vortexed for 1 min. Appropriate dilutions were plated on MRBA and plates were incubated for 3 days (31°C, dark). Incidence of *Aspergillus* species was calculated as CFU g^–1^ of sample. Isolates from plates with less than 10 putative *Aspergillus* section Flavi colonies were transferred onto 5-2 agar and incubated (5 days, 31°C, dark). *Aspergillus* isolates were assigned to their corresponding species based on colony morphology, spore ornamentation, and aflatoxin production profile (Cotty, 1989; Cotty and Cardwell, 1999; Frisvad et al., 2019). Isolates were saved as agar plugs (3-mm dia) of sporulating cultures in 4 ml vials containing 2 ml sterile distilled water and maintained at room temperature until further characterization.

### Vegetative Compatibility Analyses

Frequencies of the AAVs to which the four active ingredient isolates of the biocontrol product belong to were determined in both soils before application and grains at harvest during 2009 and 2010. Mutants of isolates (*nit*) were generated as previously described (Atehnkeng et al., 2014, 2016). Assignment of isolates to one of the four atoxigenic AAVs was conducted by performing VCA as above.

### Quantifying Aflatoxin Concentration in Crops at Harvest and After Poor Storage

For crops examined during 2009–2012 (at harvest and after simulated poor storage), aflatoxins were extracted from sub-samples (20 g ground sample) by adding 100 ml 70% methanol (Atehnkeng et al., 2008a) for maize and 100 ml 80% methanol for groundnut (Cole and Dorner, 1993). Aflatoxin extraction and quantification was conducted as previously described (Atehnkeng et al., 2008a).

For maize from commercial fields (2013–2018), aflatoxins were quantified using GIPSA-approved Neogen Reveal^®^ Q+ for Aflatoxin kit (Neogen Corp., Lansing, MI, United States). Briefly, a 20-g sub-sample was transferred into a 500 ml media bottle and 100 ml 65% ethanol was added. The mixture was shaken for 3 min using an orbital shaker at 200 rpm, allowed to settle for 3 min, and then filtered through Whatman No. 1 filter paper (Whatman International Ltd., Maidstone, United Kingdom) into a Tri-Pour^®^ beaker. Thereafter, 500 μl of sample diluent was transferred to a sample cup and 100 μl of sample filtrate was added. A 100-μl aliquot of diluted sample was transferred into a new cup and mixed thoroughly and mixed by aspiration. A new test lateral flow strip was placed in the sample cup for 6 min ensuring that the strip touched the mixture. Then the strip was removed and read either on a Neogene AccuScan^®^ Pro or Gold Reader.

### Data Analysis

Data on CFU g^–1^, *Aspergillus* species distribution, incidence of atoxigenic AAVs, and aflatoxin concentration (response variable, *x*) were transformed using the equation *y* = log_10_(1 + *x*) to stabilize the variance prior to analysis. Depending on the comparisons, means were separated using paired *t*-tests (PROC T TEST, α = 0.05) or protected Fisher’s least significant difference test (LSD, α = 0.05) using SAS software (version 9.2, SAS Institute Inc., Cary, NC, United States). Untransformed data are presented in summary tables and graphs in this paper.

## Results

### Quality of the Biocontrol Products

During the 10-year study, 382 batches of the biocontrol product were produced and examined for quality control parameters. Batches include the biocontrol formulation produced using both the laboratory- and industrial-scale processes. The quantity of the biocontrol product produced per year varied (range = 1.54 tons in 2009 to 779.42 tons in 2017). In all examined batches, 100% of carrier grains were colonized by *A. flavus*. Other microorganisms were not found in any of the examined carrier grains of any of the 382 batches. VCA revealed that the recovered fungi belonged solely to the four atoxigenic AAV active ingredients of the biocontrol product. In general, each of the four atoxigenic AAVs was found on 25 ± 8% of the carrier grains. On an average, there were 3,500 ± 500 CFU g^–1^ of product.

### Fungal Densities in Field Efficacy Trials: 2009 and 2010

Across treated and control fields, *Aspergillus* section Flavi densities ranged from 304 CFU g^–1^ to 1,050 CFU g^–1^ of soil (Table 2). In all cases, population densities in soil prior to biocontrol application were similar (*P* > 0.05) in treated and control fields. At harvest, maize and groundnut grains from treated fields contained *Aspergillus* section Flavi densities ranging from 185 CFU g^–1^ to 4,117 CFU g^–1^ compared to 72 CFU g^–1^ to 4,839 CFU g^–1^ in grains from control fields (Table 2). There were no significant (*P* > 0.05) differences in CFU g^–1^ between grains from treated and control fields.

**TABLE 2:** Colony-forming units (CFU) g^–1^ of *Aspergillus* section *Flavi* fungi in soil before biocontrol application and in maize and groundnut grains harvested from farmers’ fields that were either treated or not treated during 2009 and 2010.

**Year**	**Crop**	***n***	**Treatment**	**CFU g^–1z^**	
				
				**Soil before inoculation**	**Grain at harvest**
2009	Maize	51	Biocontrol	957^a^	1,387^a^
		51	Control	850^a^	1,325^a^
	Groundnut	8	Biocontrol	375^a^	185^a^
		8	Control	1,050^a^	72^a^
2010	Maize	14	Biocontrol	304^a^	4,117^a^
		14	Control	491^a^	4,839^a^
	Groundnut	16	Biocontrol	1,012^a^	1,963^a^
		16	Control	748^a^	2,447^a^

*^*z*^CFU g^–1^ were log transformed (log_10_[CFU g^–1^ + 1]) prior to analysis to stabilize the variance. The means of paired treated and control fields for a crop within each column with different letters are significantly different according to Student’s *t*-test (α = 0.05).*

### Distribution of *Aspergillus* Section Flavi in Field Efficacy Trials: 2009 and 2010

The native *Aspergillus* fungi found in soil were potentially high aflatoxin producers in all farmers’ fields where efficacy trials were conducted. In 2009, groundnut fields had high levels of S_BG_ strains while in 2010, maize fields had high levels of both S_BG_ strains and *A. parasiticus* before treatment (Table 3). In treated and control maize and groundnut fields, the *A. flavus* L morphotype dominated both soils before inoculation and grains at harvest. In the corresponding treated and control fields, frequencies of each fungal type were similar (*P* > 0.05) except for both the L morphotype and S_BG_ strains in treated groundnut soil during 2009 (Table 3) and treated maize grain during 2010 (Table 3). When comparing fungal types within treated or control samples, in all cases, the L morphotype had significantly (*P* > 0.05) higher frequencies than the other types (Table 3). Members of the S_BG_ strain, which produce large concentrations of B and G aflatoxins, were rarely detected in treated maize and *A. parasiticus* was never detected in treated maize or groundnut fields (Table 3). These aflatoxin producers were almost completely replaced by *A. flavus* L morphotype fungi, to which the four atoxigenic active ingredient isolates of the biocontrol product belong (Table 3).

**TABLE 3:** Frequencies of *Aspergillus* section *Flavi* fungi in soil before biocontrol application, and in maize and groundnut grains from biocontrol -treated and control fields during 2009 and 2010.

**Year**	**Crop**	***n***	**Treatment**	***Aspergillus* fungi distribution (%)^y^**							
				
				**Soil before inoculation^z^**				**Grain at harvest**			
					
				**L**	**S_BG_**	**P**	**T**	**L**	**S_BG_**	**P**	**T**
2009	Maize	51	Biocontrol	85.9^aA^	3.5^aB^	1.0^aB^	9.6^aB^	99.7^aA^	0.3^aB^	0.0^aB^	0.0^aB^
		51	Control	87.4^aA^	2.7^aB^	1.7^aB^	8.2^aB^	93.3^aA^	5.9^aB^	0.5^aB^	0.3^aB^
	Groundnut	8	Biocontrol	99.2^aA^	0.5^bB^	0.3^aB^	0.0^aB^	100.0^aA^	0.0^aB^	0.0^aB^	0.0^aB^
		8	Control	82.4^bA^	11.5^aB^	0.3^aB^	5.8^aB^	95.6^aA^	1.6^aB^	2.8^aB^	0.0^aB^
2010	Maize	14	Biocontrol	89.9^aA^	7.1^aB^	3.0^aB^	–	100.0^aA^	0.0^bB^	0.0^aB^	–
		14	Control	77.9^aA^	14.8^aB^	7.3^aB^	–	83.4^bA^	16.3^aB^	0.3^aC^	–
	Groundnut	16	Biocontrol	97.8^aA^	2.2^aB^	0.0^aB^	–	100.0^aA^	0.0^aB^	0.0^aB^	–
		16	Control	96.3^aA^	2.8^aB^	0.9^aB^	–	95.7^aA^	3.5^aB^	0.8^aB^	–

*^*y*^The frequency (%) of fungi (*x*) were log transformed (log_10_[*x* + 1]) prior to analysis to stabilize the variance. L, *A. flavus* L morphotype; S_*BG*_, S_*BG*_ strains; P, *A. parasiticus*; T = *A. tamarii* (not found in 2010). ^*z*^The means of paired treated and control fields for a crop within each column with different lowercase letters are significantly different (Student’s *t*-test, α = 0.05). Means of fungi within a row with different uppercase letters are significantly different (Fisher’s LSD test, α = 0.05).*

### Vegetative Compatibility Analyses in Samples From Field Efficacy Trials: 2009 and 2010

In both 2009 and 2010, frequencies of the biocontrol active ingredient AAVs in soils before inoculation were not significantly (*P* > 0.05) different between fields to be treated (range = 1.1–6.5%) and their respective control fields (range = 0.7–7.8%) (Table 4). The observed frequencies prior to product application demonstrate that the biocontrol active ingredient AAVs are relatively common across the major maize and groundnut producing regions of Nigeria.

**TABLE 4:** Incidence of atoxigenic African *Aspergillus flavus* vegetative compatibility groups (AAVs) active ingredients of a biocontrol product in soil before its application, and maize and groundnut grains from treated fields and control fields during 2009 and 2010.

**Year**	**Crop**	**Treatment**	**Biocontrol AAVs (%)^y^**	
			
			**Soil before inoculation^z^**	**Grain at harvest**
2009	Maize	Biocontrol	1.1^aB^	75.9^aA^
		Control	0.7^aA^	11.6^bA^
	Groundnut	Biocontrol	6.5^aB^	71.9^aA^
		Control	7.8^aA^	4.7^bA^
2010	Maize	Biocontrol	3.9^aB^	68.8^aA^
		Control	3.5^aA^	2.5^bA^
	Groundnut	Biocontrol	4.8^aB^	83.8^aA^
		Control	2.3^aA^	3.7^bA^

*^*y*^Percentage of the four atoxigenic active ingredient AAVs = [(*y* × 100)/n] where *y*, number of isolates belonging to any of the four AAVs, and n, total number of *A. flavus* L morphotype isolates. Isolates were assigned to AAVs using vegetative compatibility analysis. ^*z*^The means of paired treated and control fields for a crop within each column with different lowercase letters are significantly different (Student’s *t*-test, α = 0.05). Means of the AAVs within the row with different uppercase letters are significantly different (Student’s *t*-test, α = 0.05).*

Frequencies of the biocontrol product active ingredient AAVs were always higher (*P* < 0.05) in treated crops (range = 68.8–83.8%) than in control crops (range = 2.5–11.6%). In treated fields, the frequencies of atoxigenic AAVs in soil before inoculation were always lower (*P* < 0.05) than in grains at harvest (Table 4). In contrast, in all control fields, frequencies of atoxigenic AAVs were similar (*P* > 0.05) in soils before inoculation and grains at harvest (Table 4).

Frequencies did not differ significantly among the biocontrol product AAVs in soils before treatment in control plots (Table 5). There were, however, some differences (*P* < 0.05) among atoxigenic AAV frequencies in maize soil to be treated during both 2009 and 2010 and groundnut soil to be treated during 2010 (Table 5). Ka16127 had higher frequencies than the other active ingredients in maize soil during 2009. Both La3279 and Ka16127 had higher frequencies than the other two atoxigenic AAVs in both maize and groundnut soil during 2010. In grains of treated fields, both La3279 and Ka16127 generally dominated communities of both crops during both years (Table 5). In grain from control fields, La3279 had higher frequencies in maize (2009) and groundnut (2010); Ka16127 and La3279 had similar frequencies in groundnut (2010) (Table 5).

**TABLE 5:** Incidence (%) of the four atoxigenic African *Aspergillus flavus* vegetative compatibility groups (AAVs) constituting an aflatoxin biocontrol product in soil before its application and in maize and groundnut grains from treated and control fields during 2009 and 2010.

**Year**	**Crop**	**AAVs**	**Incidence (%) of AAV^y^**			
			
			**Soil before**		**Grain at harvest**	
			**treatment^z^**			
				
			**Treated**	**Control**	**Treated**	**Control**
2009	Maize	La3279	0.4^b^	0.0^a^	31.9^a^	7.0^a^
		Ka16127	1.1^a^	0.4^a^	20.3^b^	1.7^b^
		La3304	0.2^b^	0.4^a^	12.5^bc^	1.8^b^
		Og0222	0.1^b^	0.1^a^	9.1^c^	1.2^b^
	Groundnut	La3279	3.9^a^	3.1^a^	29.7^a^	1.6^a^
		Ka16127	0.8^a^	3.1^a^	19.0^ab^	0.8^a^
		La3304	0.1^a^	0.8^a^	7.0^b^	1.6^a^
		Og0222	0.8^a^	0.8^a^	15.6^ab^	0.8^a^
2010	Maize	La3279	1.8^a^	0.9^a^	29.0^a^	0.9^a^
		Ka16127	1.3^ab^	0.4^a^	21.4^ab^	1.3^a^
		La3304	0.9^b^	0.0^a^	16.1^ab^	0.9^a^
		Og0222	0.0^b^	0.0^a^	7.6^b^	0.4^a^
	Groundnut	La3279	1.6^ab^	0.4^a^	35.2^a^	4.3^a^
		Ka16127	2.7^a^	0.8^a^	29.7^a^	2.3^ab^
		La3304	0.4^b^	0.0^a^	5.1^b^	0.4^b^
		Og0222	0.0^b^	0.0^a^	3.5^b^	0.4^b^

*^*y*^Percentage of AAVs = [(*y* × 100)/n] where *y*, number of isolates belonging to the specific AAVs, and n, total number of *A. flavus* L morphotype isolates. Isolates were assigned to AAVs using vegetative compatibility analysis. ^*z*^The means of paired treated and control fields for a crop within each column with different lowercase letters are significantly different (Fisher’s LSD test, α = 0.05).*

### Aflatoxin Concentrations in Grain at Harvest and After Poor Storage

At harvest, treated crops from field efficacy trials (2009–2012) generally contained low aflatoxin content and significantly (*P* < 0.05) less aflatoxins (82–95% less) than untreated crops (Table 6). The only case in which the average of treated crops contained >20 ppb total aflatoxin was maize in 2010 (21 ppb). However, during that year, average aflatoxin content in maize from the 14 examined control fields was 372.4 ppb (Table 6), a high and unsafe level.

**TABLE 6:** Total aflatoxin concentration in freshly harvested and poorly stored maize and groundnut grains from biocontrol-treated and control fields in Nigeria during 2009 to 2012.

**Crop and its stage**	**Treatment**	**Aflatoxin (ppb)**							
		
		**2009**		**2010**		**2011**		**2012**	
					
		**Mean^x^**	**Red (%)^y^**	**Mean**	**Red (%)**	**Mean**	**Red (%)**	**Mean**	**Red (%)**
**At harvest**									
Maize	Biocontrol	2.7^b^	82	21.0^b^	94	3.7^b^	83	1.8^b^	86
	Control	14.8^a^		372.4^a^		22.3^a^		12.9^a^	
Groundnut	Biocontrol	0.0	–	2.7^b^	95	3.1^b^	85	–	–
	Control	0.0		54.6^a^		20.3^a^		–	
**After simulated poor storage**									
Maize	Biocontrol	18.4^b^	82	26.2^b^	93	25.8^b^	89	50.3^b^	90
	Control	245.1^a^		399.0^a^		238.2^a^		527.4^a^	
Groundnut	Biocontrol	–^z^	–	9.5^b^	80	29.9^b^	80	–	–
	Control	–		47.5^a^		152.4^a^		–	

*^*x*^The means of paired treated and control fields for a crop within each column with different letters are significantly different according to the Student’s *t*-test (α = 0.05). Mean aflatoxin concentration in grains from maize fields (51 in 2009, 14 in 2010, 199 in 2011, and 38 in 2012) and groundnut fields (8 in 2009, 16 in 2010, and 82 in 2011). ^*y*^Red denotes aflatoxin reduction (%) = [1 – (aflatoxin in treated sample)/(aflatoxin in control sample)] × 100. ^*z*^Not tested.*

When crops were subjected to simulated poor storage conditions, the protection provided by biocontrol continued, with treated crops accumulating 80% to 100% less aflatoxins than untreated crops (Table 6). Aflatoxin content in untreated crops increased dramatically due to simulated poor storage conditions. For example, the total aflatoxin content in maize at harvest from 2009 was 14.8 ppb and after simulated poor storage it increased to 245.1 ppb. Large increases in aflatoxin content occurred in the other poorly stored control grains, with up to 40-fold aflatoxin increase (maize 2012; Table 6).

In commercial maize grain samples, there were 72–94% less aflatoxin in treated maize compared to controls during 2015, 2017, and 2018 (Table 7). During years when aflatoxin levels were <10 ppb in untreated grains (2014 and 2015), aflatoxin reductions provided by the biocontrol were less pronounced (72–76%) compared to years when untreated maize contained high aflatoxin levels (88–94%; Table 7). A few samples with high aflatoxin level were encountered among treated samples in 2016 and 2017 (Table 7). Variance of aflatoxin concentration in grain samples from treated fields was 53% (in 2014) to 99% (in 2018) lower than samples from control fields (Table 7). Although aflatoxin concentration was measured in grains from treated fields in 2013 and 2016, aflatoxin reduction could not be quantified due to lack of samples from control fields.

**TABLE 7:** Aflatoxin concentration in samples from commercial maize grain aggregated from biocontrol-treated and control fields by commercial enterprises in Nigeria during 2013–2018.

**Year**	**Treatment**	**Min (ppb)**	**Max (ppb)**	**Variance**	**Mean (ppb)^x^**	**Reduction (%)^y^**
2013	Treated	0.0	70	11	0.5	–
	Control	–^z^	–	–	–	
2014	Treated	0.0	141	108	1.7^b^	72
	Control	0.0	103	233	6.1^a^	
2015	Treated	0.0	134	106	2.4^b^	76
	Control	0.1	147	711	9.7^a^	
2016	Treated	0.0	1,094	6,347	16.9	–
	Control	–	–	–	–	
2017	Treated	0.0	870	473	3.6^b^	88
	Control	0.0	1,971	17,995	29.6^a^	
2018	Treated	0.0	174	144	3.3^b^	94
	Control	0.0	738	11,348	55.3^a^	

*^*x*^The mean of aflatoxin concentration in sampled grains from treated (660 in 2013; 213 in 2014; 292 in 2015; 1,314 in 2016; 2,451 in 2017; and 2,751 in 2018); and untreated maize samples (99 in 2014, 109 in 2015, 257 in 2017, and 240 in 2018). The means of treated and control pairs for any year within each column with different letters are significantly different according to the Student’s *t*-test (α = 0.05). ^*y*^Aflatoxin reduction (%) = [1 – (aflatoxin in treated sample)/(aflatoxin in control sample)] × 100. ^*z*^Not tested.*

### Samples Meeting Standards

Treated crops in efficacy trials had higher proportions of samples below the European Union 4 ppb maximum allowable level for total aflatoxins both at harvest (2009–2012) and after simulated poor storage (2009 and 2010) compared to controls (Table 8). In 2009, 2010, and 2012, none of the treated crops exceeded the US action level of 20 ppb total aflatoxins at harvest. In 2011, only 6.5% of the treated maize and 9.8% of the treated groundnut had >20 ppb total aflatoxins at harvest (Table 8), even though during that year high total aflatoxin content occurred in both control maize (avg. = 372.4 ppb) and control groundnut (avg. = 54.6 ppb) (Table 6). Also see Supplementary Table 2.

**TABLE 8:** Proportion of farmers meeting various total aflatoxin standards in freshly harvested and poorly stored maize and groundnut grains from farmers’ fields that were either treated or not treated (control) with a biocontrol product in Nigeria during 2009 to 2012^u^.

**Crop and its stage^v^**	**Aflatoxin content (ppb)^w^**	**Proportion of farmers’ fields (%)^x^**							
		
		**2009**		**2010**		**2011**		**2012**	
					
		**Biocontrol**	**Control**	**Biocontrol**	**Control**	**Biocontrol**	**Control**	**Biocontrol**	**Control**
**At harvest**									
Maize	<4	76.5^a^	29.4^b^	50.0^a^	0.0^b^	50.8^a^	12.6^b^	78.9^a^	52.6^b^
	<10^y^	96.1^a^	54.9^a^	64.3^a^	21.4^b^	71.9^a^	30.7^b^	94.7^a^	71.1^b^
	<20^y^	100.0^a^	72.5^a^	100.0^a^	35.7^b^	93.5^a^	70.9^b^	100.0^a^	76.9^b^
	>20	0.0^b^	27.5^a^	0.0^b^	64.3^a^	6.5^b^	29.1^a^	0.0^b^	23.1^a^
Groundnut	<4	100.0^a^	100.0^a^	62.5^a^	68.8^a^	50.0^a^	22.0^b^	–	–
	<10	0.0	0.0	81.3^a^	87.5^a^	75.6^a^	41.5^b^	–	–
	<20	0.0	0.0	100.0^a^	93.8^a^	90.2^a^	58.5^b^	–	–
	>20	0.0	0.0	0.0^a^	6.2^a^	9.8^b^	41.5^a^	–	–
**After simulated poor storage**									
Maize	<4	31.4^a^	0.0^b^	21.4^a^	0.0^b^	31.3^a^	5.5^b^	7.9^a^	0.0^b^
	<10	56.9^a^	0.0^b^	57.1^a^	9.7^b^	44.5^a^	10.9^b^	10.5^a^	0.0^b^
	<20	70.6^a^	3.9^b^	71.4^a^	28.6^b^	63.3^a^	18.0^b^	26.3^a^	0.0^b^
	>20	29.4^b^	96.1^a^	28.6^b^	71.4^a^	36.7^b^	82.0^a^	73.7^b^	100.0^a^
Groundnut	<4	–^z^	–	73.3^a^	40.0^b^	75.8^a^	28.8^b^	–	–
	<10	–	–	86.7^a^	40.0^b^	83.3^a^	37.9^b^	–	–
	<20	–	–	93.3^a^	73.3^b^	87.9^a^	43.9^b^	–	–
	>20	–	–	6.7^b^	26.7^a^	12.1^b^	56.1^a^	–	–

*^*u*^For maize, the number of pairs of treated and control fields were 51 in 2009, 14 in 2010, 199 in 2011, and 38 in 2012. The respective numbers for groundnut were 8 in 2009, 16 in 2010, and 82 in 2011. ^*v*^Maize grain and groundnut kernel samples for aflatoxin analysis were either processed immediately after harvest or subjected to simulated poor storage conditions. ^*w*^<4 ppb is the European Union maximum total aflatoxin limit for human consumption; <10 ppb is the World Food Program maximum total aflatoxin limit; <20 ppb is the United States Food and Drug Administration action level for total aflatoxins in food; and >20 ppb is universally considered unacceptable for human consumption. ^*x*^The means of paired treated and control fields of a crop for a year within each row with different letters are significantly different according to the Student’s *t*-test (α = 0.05). ^*y*^The number of samples within a category was divided by total number of samples and then the quotient was divided by 100 to calculate the percentages. Note that the number of samples having <4 ppb aflatoxins were added to the number of samples having 4 to <10 ppb aflatoxins to derive the number of samples with <10 ppb aflatoxins. Similarly, the counts for <20 ppb also contained counts for samples with <10 ppb, and thus, also counts for samples with <4 ppb. ^*z*^Groundnut not examined during the year/stage.*

After simulated poor storage, frequencies of biocontrol-treated crops containing <4 ppb total aflatoxins ranged from 7.9 to 31.4% while for untreated crops the range was 0–5.5% (Table 8). The proportion of crops containing >20 ppb total aflatoxins ranged from 28.6 to 73.7% for treated crops and from 71.4 to 100% for control crops (Table 8).

For commercially produced maize treated with the biocontrol product during the 2013 to 2018 seasons, the proportion of samples containing <4 ppb total aflatoxins ranged from 65.8 to 98.5% (Table 9) with only 0.6–9.7% having >20 ppb total aflatoxins. The proportion of crops from control fields examined in 2014, 2015, 2017, and 2018 containing <4 ppb total aflatoxins were 81, 84, 43, and 25%, respectively. The proportion of control samples containing >20 ppb total aflatoxins in the same years were 9.1, 11.9, 23.0, and 37.1%, respectively (Table 9). Also see Supplementary Table 3.

**TABLE 9:** Proportion of samples meeting various total aflatoxin standards in freshly harvested maize grain from farmers’ fields that were treated commercially with a biocontrol product in Nigeria during 2013–2018 and from control fields in nearby locations that did not apply the product.

**Aflatoxin content (ppb)^x^**	**Proportion of samples (%)^w^**											
	
	**2013**		**2014**		**2015**		**2016**		**2017**		**2018**	
						
	**Biocontrol**	**Control**	**Biocontrol**	**Control**	**Biocontrol**	**Control**	**Biocontrol**	**Control**	**Biocontrol**	**Control**	**Biocontrol**	**Control**
<4^y^	98.5	–^z^	93.9	80.8	94.9	84.4	65.8	–	89.7	43.2	87.9	24.6
<10	99.1	–	96.2	82.8	96.9	84.4	85.6	–	93.7	49.8	94.0	50.0
<20	99.4	–	98.6	90.9	98.6	88.1	90.3	–	96.5	77.0	97.3	62.9
>20	0.6	–	1.4	9.1	1.4	11.9	9.7	–	3.5	23.0	2.7	37.1

*^*w*^The number of examined samples were 660 in 2013; 213 in 2014; 292 in 2015; 1,314 in 2016; 2,451 in 2017; and 2,751 in 2018. The respective numbers for untreated samples were 99 in 2014, 109 in 2015, 257 in 2017, and 240 in 2018. In 2013, 660 samples were collected from stores of individual farmers’ fields soon after harvest. Each sample during 2014–2018 represent a grain lot of approximately 30 tons aggregated by commercial enterprises from farmers. ^*x*^<4 ppb is the European Union maximum total aflatoxin limit for human consumption; <10 ppb is the World Food Program maximum total aflatoxin limit; <20 ppb is the United States Food and Drug Administration action level for total aflatoxins in food; and >20 ppb is universally considered unacceptable for human consumption. Maize grain samples for aflatoxin analysis were processed shortly post-harvest after aggregation. ^*y*^The number of samples within a category was divided by total number of samples and then the quotient was divided by 100 to calculate the percentages. Note that the number of samples having < 4 ppb aflatoxins were added to the number of samples having 4 to <10 ppb aflatoxins to derive the number of samples with <10 ppb aflatoxins. Similarly, the counts for <20 ppb also contained counts for samples with <10 ppb, and thus, also counts for samples with <4 ppb. ^*z*^Samples from control fields not collected in 2013 and 2016.*

## Discussion

The current study provides a decade-long summary of efficacy of the aflatoxin biocontrol product Aflasafe^®^ in maize and groundnut cropped in Nigeria. Aflasafe^®^ is the first aflatoxin biocontrol product registered for use in any African nation and the third worldwide. The 10-year record provides substantial evidence of the stability of the biocontrol product efficacy in limiting aflatoxin content in farmers’ fields across the diverse cropping systems of Nigeria within fluctuating and sometimes challenging environmental conditions. Biocontrol-treated crops became contaminated with significantly less aflatoxins than untreated crops. Most of the treated crops met the stringent tolerance thresholds of local and international food and feed premium markets. A significant proportion (94%) of the 7,681 biocontrol-treated commercial crop samples that were examined had less than the World Food Program maximum limit of 10 ppb total aflatoxins. Initially (2009–2012), biocontrol efficacy was evaluated in a relatively modest number of maize and groundnut fields (14–199 for maize; 8–82 for groundnut; total of 408 treated and 408 control fields). However, the number of fields and the size of each field (∼1 ha) during the 2009–2012 efficacy evaluations were considerably larger than other reported biocontrol efficacy studies that used fewer than five replicates per year with some plots less than 0.01 ha (Alaniz Zanon et al., 2013, 2016; Pitt et al., 2015; Weaver et al., 2015; Molo et al., 2019; Weaver and Abbas, 2019). After the testing and refinement phase, aflatoxin biocontrol usage in Nigeria grew exponentially through the AgResults Project (2013–2018) in which thousands of farmers used biocontrol and other good agricultural practices. Over 210,000 tons of maize with acceptable aflatoxin concentrations (<10 ppb) were produced through the AgResults Project. A large portion of the commercially produced biocontrol-treated maize entered premium markets in Nigeria while the other portion was either consumed by the farmers and their families or sold in local, informal markets (Schreurs et al., 2019). Reduced aflatoxin exposure brought health benefits for maize famers and consumers, and economic benefits for farmers and associated industries (Narayan et al., 2019).

Several crops grown in Nigeria frequently become contaminated with unsafe aflatoxin concentrations (Bandyopadhyay et al., 2007; Singh and Cotty, 2017; Ezekiel et al., 2018, 2019; JECFA, 2018) leading to unacceptable aflatoxin exposure in humans (Ezekiel et al., 2014). This negatively impacts health, income, and trade. One example is lack of access of Nigerian groundnuts to European markets due to high aflatoxin levels. Agricultural, climatic, cultural, infrastructural, and institutional practices and/or conditions all contribute to aflatoxin contamination (Bandyopadhyay et al., 2016). Therefore, aflatoxin management is best with a holistic approach (Ayalew et al., 2017; Logrieco et al., 2018). Intervention during the initial stages of crop infection by aflatoxin producers is a desired component of such strategies and use of atoxigenic *A. flavus* as biocontrol agents during crop development is effective (Cotty and Mellon, 2006).

### Improvement in Biocontrol

The atoxigenic biocontrol technology developed by USDA-ARS uses a single atoxigenic genotype as active ingredient fungus (Cotty et al., 2007). This strategy protects crops from field to plate because after sporulating in the field and becoming associated with the treated crop, the beneficial fungi move with the crops to storage and continue to prevent aflatoxin production should conditions for production again become favorable (Mehl et al., 2012; Atehnkeng et al., 2014; Bandyopadhyay et al., 2016; Senghor et al., 2019). The adaptation and improvement of the technology for use in SSA included use of four atoxigenic AAVs as active ingredient fungi rather than a single isolate (Bandyopadhyay et al., 2016). Use of multiple atoxigenic isolates belonging to diverse AAVs may provide longer-term protection compared to using single genotypes (Probst et al., 2011; Mehl et al., 2012). In the current study, four atoxigenic AAVs native to Nigeria were used in a biocontrol formulation to limit maize and groundnut aflatoxin content.

### Testing Under Natural Setting and Product Registration

When IITA and USDA-ARS initiated talks with NAFDAC, the biopesticide regulator in Nigeria, to develop an aflatoxin biocontrol program for Nigeria, it was decided that the work would require applications in multiple fields covering several agro-ecologies over multiple years. Testing the biocontrol product under natural settings without researcher interventions (i.e., farmers used their traditional practices) other than farmer training in application method and provision of the biocontrol product for a single application allowed testing under real-life situations that smallholder Nigerian farmers face. A natural-setting strategy, although in large-scale commercial agriculture, was conducted during efficacy trials to register the biocontrol products AF36 (Cotty, 2006) and Afla-Guard^®^ (Dorner, 2004) in the United States (USEPA, 2003, 2004). The results from 2009 and 2010, used for preparing a dossier for registration, demonstrated that, compared to control, biocontrol application (i) did not increase fungal densities in treated crops (Table 2), (ii) significantly lowered frequencies of aflatoxin producers in treated crops (Table 3), (iii) promoted a higher incidence of atoxigenic AAVs in treated crops (Tables 4, 5), and (iv) lowered aflatoxin content in treated crops both at harvest and after simulated poor storage (Tables 6, 7). Because of these results, in 2014 NAFDAC approved the unrestricted use of Aflasafe^®^ for aflatoxin mitigation in maize and groundnut throughout Nigeria. The registration allowed thousands of smallholder farmers in Nigeria to use biocontrol commercially for producing aflatoxin standard-compliant maize. In addition, the biocontrol product has also been proven to effectively limit aflatoxin contamination of chili peppers although its registration with NAFDAC for unrestricted use in chili peppers is still pending (Ezekiel et al., 2019).

### *Aspergillus* Community Modulation by Biocontrol

In treated crops, *A. flavus* L morphotype predominated, and most of the L morphotype isolates belonged to the atoxigenic active ingredient AAVs of the biocontrol product (Table 4). The high frequency of atoxigenic AAVs in grains from treated crops illustrate excellent competitiveness for displacing aflatoxin producers. Across substrates and years, AAVs La3279 and Ka16127 were consistently the most commonly recovered genotypes in treated fields (Table 5). La3279 and Ka16127 were also the most commonly recovered genotypes from treated fields of the preliminary experiments (Atehnkeng et al., 2014). Both genotypes appear to be more competitive than the two other atoxigenic AAVs composing the biocontrol product. Some of the factors that affect the competitiveness of biocontrol isolates are differential ability to infect and colonize hosts/debris, produce spores, disperse from soil to crop, cause secondary infection, among others (Mehl et al., 2012). The observed low aflatoxin levels in the treated crops are a direct consequence of the almost complete replacement of high aflatoxin producers by the atoxigenic AAVs composing the biocontrol product. Even when S_BG_ strains and *A. parasiticus* occur at a relatively low frequency, as in the control crops (range = 0.3–16.3%; Table 3), aflatoxin levels can reach to over 300 ppb (maize in 2010; Table 6). Therefore, the biocontrol application promoted communities with less aflatoxin-producing potential and consequently lower aflatoxin levels were detected in treated crops both at harvest and after the simulated poor storage.

One of the advantages of changing the *Aspergillus* community structure in favor of the applied atoxigenic AAVs is that the safer *Aspergillus* community structure translates to not only nearly uniform low aflatoxin levels but also reduced variance in aflatoxin concentration in grains. Relatively recently, it was reported that Aflasafe SN01^®^ usage in Senegal resulted in reduced variance in aflatoxin content of treated crops in efficacy trials in farmers’ fields in groundnut and maize (Senghor et al., 2019). Our studies on maize in commercial fields in Nigeria confirmed the previous observations in Senegal. Low variance in aflatoxin content indicates that sample to sample variations in aflatoxin concentration are low. This reduces the risk that lots measured as low in aflatoxin would actually have unacceptable aflatoxin content and be rejected after receipt in a location with high value markets. Thus, biocontrol treatments might be expected to reduce both aflatoxin-concentration heterogeneity and costs associated with shipment of crops that ultimately become rejected and destroyed or turned away at the destination. High variance in control grain lots suggests significant risk of undetected aflatoxin contamination in the absence of biocontrol treatment. Maximum aflatoxin level in a sample from treated maize reached 1,094 ppb in a grain lot of 2016. Thus, although the sample was considered as treated, the high aflatoxin content suggests that some or few of the farmers that contributed to that 30-ton lot did not apply the biocontrol product at the indicated rate and/or at the mandatory maize growth stage (2-to-3 weeks before flowering).

### Aflatoxin Reduction by Biocontrol

Even though use of biocontrol results in excellent aflatoxin reductions at harvest and after storage (Tables 6–9), there were cases in which the protection provided by biocontrol was insufficient to limit aflatoxin content to below tolerance thresholds. For example, treated maize in 2010 had an average total aflatoxin content of 21 ppb (Table 6) and, in 2016, 10% of treated samples contained >20 ppb total aflatoxins (Table 9). In addition, if treated crops were stored poorly, aflatoxin content, although dramatically lower than poorly stored control crops, may increase above tolerance thresholds (Table 6). Therefore, it is critical to use biocontrol along with all other available, practical management practices. Relatively recently, use of biocontrol without other intervention was considered a pitfall (Njoroge, 2018). One of the objectives of the preliminary experiments (Atehnkeng et al., 2008b, 2014) and of the field efficacy trials reported in the current study was to demonstrate the value of biocontrol with no other intervention. This allowed determining the extent of protection by biocontrol alone. Aflatoxin reduction by biocontrol was prominent when aflatoxin pressure was high. In years when biocontrol efficacy resulted in less than 80% reduction, the aflatoxin content in untreated crops was low (<10 ppb total aflatoxin). Therefore, the efficacy of biocontrol in those years was not as obvious as in years where higher aflatoxin concentrations occurred in control crops (Table 6). Our investigations during 2009 to 2012 revealed that the atoxigenic AAVs of the biocontrol product are effective at limiting contamination well over 70% and up to 100% even in the absence of improved agronomic and storage practices. Certainly, if other management strategies were used during both the initial studies (Atehnkeng et al., 2008b, 2014) and the farmer-field efficacy trials (2009–2012) reported in the current study, the aflatoxin concentrations both at harvest and after storage would have been even lower, especially after storage since at harvest most treated crops contained low total aflatoxin levels.

Protection provided by atoxigenic genotypes during post-harvest conditions has been questioned (Villers, 2014; Ehrlich et al., 2015; Gressel and Polturak, 2018). However, treated crops become associated with high frequencies of the applied fungi before harvest (Tables 4, 5). If conditions for fungal infection and aflatoxin formation occur during storage, most *Aspergillus* fungi growing in stored treated crops would be the applied atoxigenic isolates. Less aflatoxin content occurred in treated crops compared to untreated crops (Table 6) and this is attributed to high frequencies of atoxigenic AAVs composing the biocontrol product in the treated crops (Tables 4, 5). Thus, treating crops with the biocontrol product during crop development provided protection during deliberately poor post-harvest storage (Table 6). Post-harvest benefits when treating groundnut with Aflasafe SN01^®^ in Senegal (Senghor et al., 2019) and maize with an experimental biocontrol formulation in Nigeria (Atehnkeng et al., 2014) have been reported.

### Paradigm Shifts in Perception of Biocontrol in Africa

There is the notion that in SSA, and consequently in Nigeria, biocontrol usage has not gone beyond the research stage because smallholder farmers cannot afford biocontrol and there is no incentive to use the technology since aflatoxin contamination is not a factor that determines price competitiveness of grains in market (Njoroge, 2018; Stepman, 2018; Pitt, 2019). In general, these are indeed challenges for scaling up any aflatoxin mitigation technology, including biocontrol, to reach thousands of farmers. Nevertheless, through diverse innovative mechanisms and partnership arrangements with various stakeholders, large-scale use of biocontrol and other aflatoxin management interventions has reached thousands of farmers in Nigeria by implementing strategies for sustainable biocontrol use through the AgResults Project and other initiatives (Bandyopadhyay et al., 2016; AgResults, 2019; Schreurs et al., 2019). The strategies include awareness and sensitization campaigns, use of improved agronomic practices, improved pre-harvest practices, use of the aflatoxin biocontrol product, improved harvest and post-harvest practices, sorting of moldy/diseased grains, proper storage and use of hermetic bags, testing, market development, policies, and any other novel, practical, and available management tool for farmers. These holistic interventions have helped to create markets willing to pay for aflatoxin standard-compliant maize (Johnson et al., 2018, 2019) resulting in farmers’ willingness to pay $11-19/ha for biocontrol (Ayedun et al., 2017). Farm-based agricultural enterprises have enabled thousands of farmers to adopt aflatoxin management strategies, centered in biocontrol, to produce and commercialize more than 200,000 tons of aflatoxin-compliant maize demonstrating that sustainability and scaling of the technology is possible. It is necessary to increase awareness about aflatoxin and create new market opportunities for aflatoxin-compliant crops for farmers to further enhance adoption of aflatoxin-mitigation practices, including biocontrol.

Biocontrol applications of atoxigenic strains have long-term carryover effects and as a result additive benefits accrue as the product is used over multiple years (Cotty et al., 2007). However, because aflatoxins influence human health managing the toxins to the lowest possible level is desired, and it is recommended that the products be applied each season maize or groundnut are produced. Costs associated with applying biocontrol products every crop cycle have been viewed as a negative aspect of the biocontrol technology (Ehrlich et al., 2015; Njoroge, 2018; Molo et al., 2019). However, optimal crop production requires application of inputs (e.g., certified seeds, fertilizers, insecticides) on a yearly basis. All of these critical inputs must be applied each cropping season and it should not be expected that atoxigenic genotypes must replace toxigenic strains with a single application. Farmers producing commercial crops in Nigeria and other SSA nations utilize inputs to increase crop production and many are now using biocontrol as a necessary input to protect crops and access premium markets. This is similar to what has occurred in high-risk portions of Texas where use of aflatoxin biocontrol products is considered a necessary cost of maize production.

Long-term studies on the efficacy and stability of aflatoxin biocontrol agents have been recommended to determine the true value of the technology in SSA (Kagot et al., 2019). It takes over a decade to develop aflatoxin biocontrol programs because it is necessary to sensitize farmers and several key stakeholders, screen for appropriate biocontrol agents, test their efficacy in multiple years, multiple areas, in real-farming conditions, develop delivery methods, register the biocontrol product with national authorities, develop commercialization strategies, establish market entry strategies, create sustainable models for biocontrol adoption in a nation-wide manner, develop infrastructure to manufacture and distribute the biocontrol product en masse, among other actions (Mehl et al., 2012; Bandyopadhyay et al., 2016; Ortega-Beltran and Bandyopadhyay, 2019; Schreurs et al., 2019). We are hopeful that the substantial evidence of the benefits of integrated management strategies centered on biocontrol products presented in this long-term study may change certain ambiguous and sometimes negative perceptions about the effectiveness of biocontrol in African contexts, its adoption, and its sustainability.

## Conclusion

Our results demonstrate that an aflatoxin biocontrol product registered for use in maize and groundnut in Nigeria, is a practical, cost-effective, and environmentally safe aflatoxin mitigation tool that enables farmers in Nigeria to produce both crops with little to no aflatoxin content. This work is the most extensive published study of the long-term efficacy of any aflatoxin biocontrol product. The results indicate that biocontrol is a stable, effective aflatoxin management tool regardless of environment or cropping system and is highly effective on crops grown by smallholder farmers. In addition, evidence of large-scale adoption of the biocontrol product in Nigeria is provided. The results suggest that biocontrol is a preferred component of holistic aflatoxin management strategies tackling agricultural, behavioral, institutional, and policy challenges. A holistic maize and groundnut aflatoxin management strategy with biocontrol as the centerpiece is contributing to better health, increased income, and greater trading opportunities. Farmers and consumers of other susceptible crops (e.g., chili peppers, sesame, sorghum) in Nigeria and elsewhere in SSA would benefit if the technology is adapted and registered for its use in those crops.

## Data Availability Statement

The raw data supporting the conclusions of this manuscript will be made available by the authors, without undue reservation, to any qualified researcher.

## Author Contributions

RB and PC designed the overall projects from which data are derived. JA, AA, AO-B, PC, and RB contributed to the conception and design of the experiments. JA, AA, AO-B, and TF conducted the experiments and field studies, and collected and analyzed the data. AA, PC, and RB provided guidance. AO-B drafted the original manuscript. JA, TF, PC, and RB edited the manuscript. All authors read, reviewed, and approved the final manuscript. RB secured funds for the study.

## Conflict of Interest

The authors declare that the research was conducted in the absence of any commercial or financial relationships that could be construed as a potential conflict of interest.
