# Supplementary material for: “Ground-Truthing” Efficacy of Biological Control for Aflatoxin Mitigation in Farmers’ Fields in Nigeria: From Field Trials to Commercial Usage, a 10-Year Study
Source: Front Microbiol. 2019 Nov 8;10:2528. doi: 10.3389/fmicb.2019.02528 (PMC6882503; doi:10.3389/fmicb.2019.02528)
Supplement: Supplementary file 1 [file Data_Sheet_1.pdf]

SUPPLEMENTARY TABLE 1. Satellite map of a section of Maigana area of Kaduna state of Nigeria annotated with an illustrative depiction of the arrangement of four out of many paired biocontrol-treated (orange) and control (brown) farmers' fields (numbered 1 to 4). Each field was at least 500 m away from any neighboring experimental field to avoid interplot interference. Note the small size of fields of the farmers. Scale bar = 500 m.

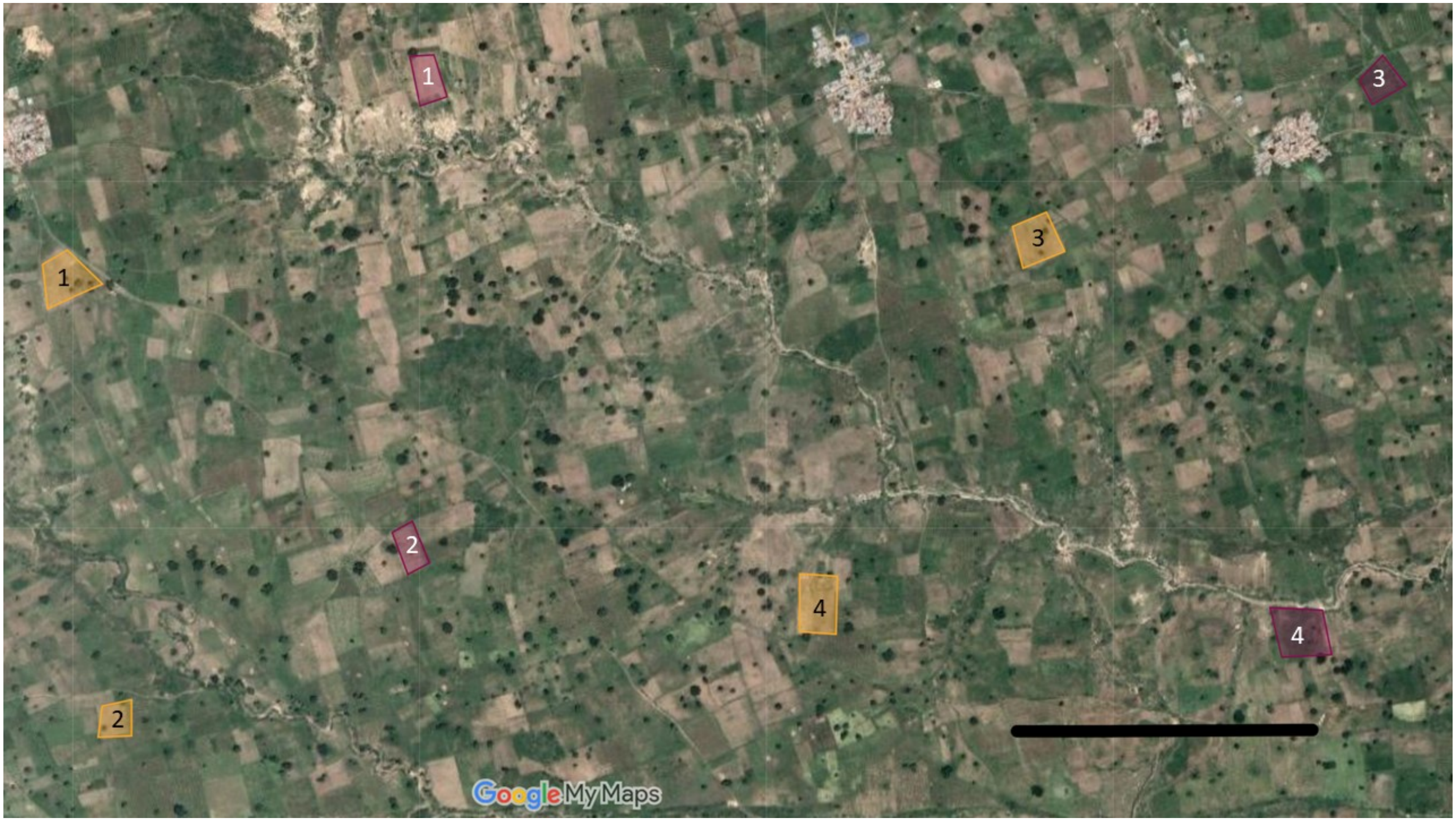

Relevant information for experimental design is given below:

Parameter	Description
Crops	Maize and groundnut.
Years	Four years: 2009 to 2012.
Location of experiments	Farmers' fields Enugu state (4 areas), Kaduna state (6 areas), Kano state (11 areas), and Oyo state (1 area).
Experimental design	Paired plot experiment.
Treatments	Biocontrol-treated and control.
Replications	Each of the 408 farmers' field treatment pair was considered as a replication. The number of pairs (or replicates) varied for crops and years. See Table 1 for the number of fields/samples pairs (= number of replications) for each crop during each year.
Size of fields	0.25 ha to 5.0 ha.
Field to field distance	500 m minimum (to avoid interplot interference of biocontrol inocula).
Cropping history of fields	Selected fields were not treated in any of the previous years and were at least 500 m away from any of the previously biocontrol treated fields (to avoid carryover of biocontrol inocula from one season to the next.

SUPPLEMENTARY TABLE 2 | Proportion of farmers meeting various total aflatoxin standards in freshly harvested and poorly stored maize and groundnut grains from farmers' fields that were either treated or not treated (control) with Aflasafe in Nigeria during 2009 to 2012

Crop and its stage[v,w]	Aflatoxin content (ppb)[x,]	Proportion of farmers' fields (%)							
		2009		2010		2011		2012	
		Aflasafe	Control	Aflasafe	Control	Aflasafe	Control	Aflasafe	Control
At harvest									
Maize	< 4 [y]	76.5	29.4	50.0	0.0	50.8	12.6	78.9	52.6
	4 to < 10	19.6	25.5	14.3	21.4	21.1	18.1	15.8	18.5
	10 to < 20	3.9	17.6	35.7	14.3	21.6	40.2	5.3	5.8
	> 20	0.0	27.5	0.0	64.3	6.5	29.1	0.0	23.1
Groundnut	< 4	100.0	100.0	62.5	68.8	50.0	22.0	-	-
	4 to < 10	0.0	0.0	18.8	18.7	25.6	19.5	-	-
	10 to < 20	0.0	0.0	18.7	6.3	14.6	17.0	-	-
	> 20	0.0	0.0	0.0	6.2	9.8	41.5	-	-
After simulated poor storage									
Maize	< 4	31.4	0.0	21.4	0.0	31.3	5.5	7.9	0.0
	4 to < 10	25.5	0.0	35.7	9.7	13.2	5.4	2.6	0.0
	10 to < 20	13.7	3.9	14.3	18.9	18.8	7.1	15.8	0.0
	> 20	29.4	96.1	28.6	71.4	36.7	82.0	73.7	100.0
Groundnut	< 4	-[z]	-	73.3	40.0	75.8	28.8	-	-
	4 to < 10	-	-	13.4	0.0	7.5	9.1	-	-
	10 to < 20	-	-	6.6	33.3	4.6	6.0	-	-
	> 20	-	-	6.7	26.7	12.1	56.1	-	-

[v] For maize, the number of pairs of treated and control fields were 51 in 2009, 14 in 2010, 199 in 2011, and 38 in 2012. The respective numbers for groundnut were 8 in 2009, 16 in 2010, and 82 in 2011.

[w] Maize grain and groundnut kernel samples for aflatoxin analysis were either processed immediately after harvest or subjected to simulated poor storage conditions.

[x] < 4 ppb is the European Union maximum total aflatoxin limit for human consumption; < 10 ppb is the World Food Program maximum total aflatoxin limit; < 20 ppb is the United States Food and Drug Administration action level for total aflatoxins in food; and > 20 ppb is universally considered unacceptable for human consumption. Maize grain samples for aflatoxin analysis were processed shortly post-harvest after aggregation.

[y] Category values were calculated independently by dividing the number of samples within a category by the total number of samples. The quotient was then multiplied by 100 to provide the percentage.

[z] Groundnut not examined in that stage/year.

SUPPLEMENTARY TABLE 3 | Proportion of samples meeting various total aflatoxin standards in freshly harvested maize grain from farmers' fields that were treated commercially with the biocontrol product Aflasafe in Nigeria during 2013 to 2018 and from untreated fields in nearby locations that did not apply Aflasafe.

Aflatoxin content (ppb)[x]	Proportion of samples (%)[w]											
	2013		2014		2015		2016		2017		2018	
	Aflasafe	Control	Aflasafe	Control	Aflasafe	Control	Aflasafe	Control	Aflasafe	Control	Aflasafe	Control
< 4[y]	98.5	-[z]	93.9	80.8	94.9	84.4	65.8	-	89.7	43.2	87.9	24.6
4 to < 10	0.6	-	2.3	2.0	2.0	0.0	19.8	-	4.0	6.6	6.1	25.4
10 to < 20	0.3	-	2.4	8.1	1.7	3.7	4.7	-	2.8	27.2	3.3	12.9
> 20	0.6	-	1.4	9.1	1.4	11.9	9.7	-	3.5	23.0	2.7	37.1

[w] The number of examined samples were 660 in 2013; 213 in 2014; 292 in 2015; 1,314 in 2016; 2,451 in 2017; and 2,751 in 2018. The respective numbers for untreated samples were 99 in 2014, 109 in 2015, 257 in 2017, and 240 in 2018. In 2013, 660 samples were collected from stores of individual farmers' fields soon after harvest. Each sample during 2014-2018 represents a grain lot of approximately 30 tons aggregated by commercial enterprises from farmers.

[x] < 4 ppb is the European Union maximum total aflatoxin limit for human consumption; < 10 ppb is the World Food Program maximum total aflatoxin limit; < 20 ppb is the United States Food and Drug Administration action level for total aflatoxins in food; and > 20 ppb is universally considered unacceptable for human consumption. Maize grain samples for aflatoxin analysis were processed shortly post-harvest after aggregation.

[y] Category values were calculated independently by dividing the number of samples within a category by the total number of samples. The quotient was then multiplied by 100 to provide the percentage.

[z] Samples from control fields not collected in 2013 and 2016.
